# Long COVID in people with mental health disorders: a scoping review

**DOI:** 10.1186/s12888-025-06935-9

**Published:** 2025-07-01

**Authors:** Catharina Münte, Manuela Glattacker, Saskia Müller, Andrea E. Zülke, Martin Heinze, Steffi G. Riedel-Heller, Dawid Pieper, Christian Jacke, Stefanie Deckert, Anne Neumann

**Affiliations:** 1 Institute for General Practice and Palliative Care, Hannover Medical School, Hannover, Germany; 2 Faculty of Health Sciences Brandenburg, Brandenburg Medical School (Theodor Fontane), Institute for Health Services and Health System Research, Rüdersdorf, Germany; 3 Center for Health Services Research, Faculty of Health Sciences, Brandenburg Medical School (Theodor Fontane), Rüdersdorf, Germany; 4 Section of Health Care Research and Rehabilitation Research, Institute of Medical Biometry and Statistics, Faculty of Medicine and Medical Center, University of Freiburg, Freiburg, Germany; 5 Bavarian Health and Food Safety Authority (LGL), State Institute of Health I:, Prevention/ Care/ Epidemiology/ E-Health, Department GP3: Bavarian Health Agency, Health Care, Nürnberg, Germany; 6 Institute of Social Medicine, Occupational Health and Public Health, Faculty of Medicine, University of Leipzig, Leipzig, Germany; 7 University Clinic for Psychiatry and Psychotherapy, Brandenburg Medical School, Immanuel Hospital Rüdersdorf, Rüdersdorf, Germany; 8 Evidence Based Practice in Brandenburg: A JBI Affiliated Group, University of Adelaide, Adelaide, Australia; 9 Scientific Institute of Private Health Insurance (WIP), Cologne, Germany; 10 Center for Evidence-Based Healthcare, Medical Faculty and University Hospital Carl Gustav Carus, TUD Dresden University of Technology, Dresden, Germany

**Keywords:** COVID-19, Long COVID, Post-Acute COVID-19 Syndrome, Mental Health, Psychiatry

## Abstract

**Background:**

Long COVID, Post COVID Syndrome or PASC (post-acute sequelae of COVID-19), according to the World Health Organization (WHO), is defined as the continuation or development of new symptoms 3 months after the initial SARS-CoV-2 infection, with these symptoms lasting for at least 2 months with no other explanation. The term Long COVID will be used throughout this review. Little is known about individuals with pre-existing mental health conditions experiencing Long COVID. This scoping review aims to provide an overview of these individuals, focusing on: 1) the course of mental disorders, 2) care needs, 3) utilization of healthcare services, and 4) psychosocial aspects, as outlined by the International Classification of Functioning (ICF).

**Methods:**

This review followed the JBI (Joanna Briggs Institute) methodology for scoping reviews and the PRISMA extension for scoping reviews. We included reports focusing on individuals with at least one pre-existing mental health diagnosis and Long COVID. Full-text reports in English or German were included, with no geographical limitations. Literature searches were conducted in PubMed, Embase, and PsycINFO on November 1, 2023, for records published between January 2020 and October 2023. Six reviewers participated in the screening process in pairs, independently conducting study selection and data extraction. Conflicts were resolved by consensus. Citation tracking was performed, and data were summarized narratively in tables.

**Results:**

From 4256 initial hits and citation tracking, 8 reports were included. The studies were heterogeneous, including chart reviews, case reports, cross-sectional, and longitudinal studies. Evidence on the impact of Long COVID on pre-existing mental health conditions was inconsistent. Most findings focused on the course of mental health disorders, ranging from symptom worsening to new symptoms of anxiety, depression, or insomnia. Evidence on mental health care needs, service utilization, and psychosocial aspects was limited.

**Conclusion:**

Limited evidence suggests that individuals with pre-existing mental health disorders who experience Long COVID may be at an increased risk of worsening mental health. However, critical aspects such as care needs, service utilization, and psychosocial factors remain under-researched, highlighting the need for further studies on mental health care for Long COVID.

**Review registration:**

Open Science Framework https://osf.io/tqexa.

**Clinical trial number:**

Not applicable.

**Supplementary Information:**

The online version contains supplementary material available at 10.1186/s12888-025-06935-9.

## Background

Severe acute respiratory syndrome coronavirus 2 (SARS-CoV-2) is responsible for the COVID-19 pandemic. COVID-19 is considered a multi-organ disease with a broad spectrum of manifestations, leading to substantial mortality and morbidity [1]. Different systems can be affected, including the respiratory, nervous, cardiovascular, gastrointestinal, musculoskeletal, neurological and endocrine systems, among others [1–4].

Following a COVID-19 infection, some individuals develop Long COVID syndrome. The WHO defines Long COVID as the continuation or development of new symptoms three months after the initial SARS-CoV-2 infection, with these symptoms lasting for at least two months and no other explanation [5].

Diagnosing Long COVID remains challenging, as while there are diagnostic tools for certain aspects of the condition, most methods are still in development [6]. Although there are attempts at a clear definition [7], Long COVID remains a diagnosis of exclusion.

Therefore, the incidence and prevalence of Long COVID cannot yet be reliably determined and vary greatly due to different study designs, settings, survey times and virus variants [2, 8–10]. In a meta-analysis and systematic review of 50 studies, the global prevalence of Long COVID was estimated at 0.43 (95% confidence interval [CI], 0.39–0.46) indicating that approximately 43% of individuals who recover from a COVID-19 infection develop long-term symptoms. The prevalence among hospitalized patients was higher at 0.54 (95% CI, 0.44–0.63), while for non-hospitalized patients, it was lower at 0.34 (95% CI, 0.25–0.46). Regional variations showed a prevalence of 0.51 (95% CI, 0.37–0.65) in Asia, 0.44 (95% CI, 0.32–0.56) in Europe, and 0.31 (95% CI, 0.21–0.43) in the United States [11]. In contrast, the World Health Organization indicates that between 10 and 20% of people infected with SARS-CoV-2, may develop Long COVID syndrome [5].

Long COVID poses substantial challenges to the healthcare system. Two key issues are particularly evident. On the one hand, there are difficulties in establishing appropriate structures for the care of individuals with Long COVID. In a study from Germany on adults with Long COVID, a majority of participants (68.3%) reported that their needs regarding treatment for Long COVID were not met [12]. Reasons expressed particularly frequently were “there were no treatment options” (46.9%), “I didn't know who to turn to” (32.4%) and “I thought I wasn't ill enough” (32.2%) [12]. On the other hand, Long COVID is associated with a notable increase in direct costs due to the increased utilization of healthcare services [13], as well as indirect costs, as individuals with the condition tend to work less and earn less than they would have otherwise [14]. A modeling study from Australia used a susceptible-exposed-infected-recovered (SEIR) model to estimate the number of people with Long COVID over time following single infections. A labor supply model was employed to estimate productivity losses as a proportion of gross domestic product (GDP). The study projected that the labor loss due to Long COVID in 2022 would amount to 102.4 million hours (0.48% of total worked hours in Australia). The estimated GDP loss from reduced labor supply and decreased use of other production factors was $9.6 billion (0.5% of GDP) [15].

The symptoms of Long COVID are diverse and encompass a wide range of physiological and psychological manifestations. Most common symptoms include fatigue, shortness of breath and cognitive impairment, as well as exhaustion, chest tightness and headaches, along with heart palpitations [6, 16]. Other studies additionally identified attention disorders, hair loss [17, 18] or mental health and pain disorders as the main symptoms, along with cardiopulmonary, neurological, gastrointestinal disorders [10]. These symptoms affect the ability to live independently, work, and perform daily activities, while also leading to financial difficulties for a substantial number of individuals [16, 19].

Long COVID symptoms are increasingly recognized not only in adults but also within the pediatric population [20]. A systematic review investigated the impact of Long COVID on children's daily lives, revealing significant limitations in their ability to participate in routine activities, such as attending school, engaging in physical exercise, and maintaining social interactions [21].

The pathogenesis of Long COVID syndrome has not yet been fully clarified but is considered to be multifactorial [2, 22]. Pathophysiological mechanisms mentioned in this context are 1) immune dysregulation, 2) viral persistence, 3) microbiome dysbiosis, 4) autoimmunity, 5) coagulopathy and endothelial dysfunction, 6) dysfunctional neurological signaling [6].

In addition to somatic symptoms, previous studies examined psychological symptoms resulting from SARS-CoV-2 infections in the context of pandemic-related stressors and persistent impairments [2, 17, 18, 21, 23–25]. There are previous studies, indicating a bidirectional relationship between a COVID-19 infection and mental health [26, 27]. Based on the known interactions between mental and physical health [28], it is essential to investigate Long COVID in this population, as pre-existing mental health conditions may exacerbate Long COVID symptoms, while Long COVID itself may further impair mental health outcomes.

Before the COVID-19 pandemic in 2019, approximately 970 million people worldwide were affected by a mental disorder, with anxiety and depression being the most prevalent [29]. Previous studies indicated that individuals with pre-existing mental health disorders are particularly vulnerable to the impact of Long COVID on their mental health as pre-existing mental health disorders are considered a risk factor for developing Long COVID [30–34]. While the pathophysiological mechanisms underlying this association are not yet fully understood, initial studies discuss that the reasons for the increased risk may lie in higher levels of inflammation and oxidative stress in individuals with psychiatric conditions, which predispose them to more severe and prolonged viral illnesses [35].

However, the current understanding of the situation of individuals with pre-existing mental health conditions and an additional Long COVID illness is still limited. We conducted a preliminary search of PubMed, PROSPERO, the Cochrane Database of Systematic Reviews and JBI Evidence Synthesis and no current or in-progress scoping reviews or systematic reviews on the topic were identified.

A synthesis of evidence on the specific situation of individuals with pre-existing mental health diagnoses and Long COVID is necessary because individuals with mental health conditions may experience altered or intensified symptoms due to Long COVID. Understanding these potential interactions could provide valuable insights into their impact on the progression of mental health disorders.

## Research question

This scoping review aims to provide an overview of the current state of research on individuals with at least one pre-existing mental health diagnosis and a Long COVID diagnosis. We consider a scoping review to be an appropriate method because the existing evidence is limited and heterogeneous, necessitating a comprehensive mapping of the available literature to identify key concepts, research gaps, and directions for future investigation.

The research question we address is: What is the current state of research regarding individuals with at least one pre-existing mental health diagnosis who are experiencing Long COVID illness, particularly in relation to the course of their mental illness, care needs, utilization of care services, and psychosocial aspects as defined by the International Classification of Functioning (ICF) [36]?

## Methods

We conducted this scoping review following the updated JBI (Joanna Briggs Institute) guidelines for scoping reviews [37]. Furthermore, we followed the ‘Preferred Reporting Items for Systematic Reviews and Meta-analysis Protocols Extension for Scoping Reviews’ (PRISMA-ScR) for reporting [38].

We emphasized the International Classification of Functioning, Disability, and Health (ICF), a framework developed by the World Health Organization (WHO) to describe health and functioning, as the central model, given its comprehensive and integrative approach to understanding health. The ICF considers not only the medical aspects of health conditions but also an individual’s functioning and contextual factors, such as personal and environmental influences [26].

By using the ICF, we integrated various perspectives on the impacts of Long COVID, including physical, mental, and social dimensions [36]. Based on the ICF, we focused on these four aspects:

### Course of the mental illness

This refers to the progression and changes in mental health conditions over time, including the onset, duration, and fluctuations in symptoms. Understanding the trajectory of mental illness is essential for effective diagnosis and treatment planning.

### Care needs

This aspect addresses the specific support and interventions required by individuals with mental health conditions. It encompasses both clinical and non-clinical needs, including medical treatment, therapy, rehabilitation, and support for daily living activities.

### Utilization of care services

This examines how individuals access and use various healthcare services, including mental health professionals, community support, and emergency care. It highlights patterns of service use and potential barriers to accessing appropriate care.

### Psychosocial aspects

This component focuses on the social and psychological factors that influence an individual's mental health, such as relationships, community engagement, and socio-economic status. It recognizes the interplay between personal experiences and broader societal influences on mental well-being.

## Eligibility criteria

### Inclusion criteria

We considered all reports that met the following eligibility criteria:

- Individuals with at least one pre-existing mental health diagnosis (according to ICD-10-GM F00-F99 or DSM)
- and who have an additional long-term/post COVID/Long COVID condition.
- Topics:
  1. Course of the mental disorder
  2. Care needs of mental disorder
  3. Utilization of care services
  4. Psychosocial aspects (according to ICF)
- Type of source: Full-text reports, no restrictions regarding study design
- Language: German and English
- Timeframe: January 2020 to October 2023
- Geographical area: no restriction

Table 1 illustrates the population, the concept, and the context considered in this scoping review.

**Table 1:** Population, concept and context

Population	Children and adults with mental and behavioral disorders (diagnosis according to ICD-10-GM F00-F99 or corresponding DSM AND Long COVID disease (WHO diagnosis code U09.9 Post-COVID-19 condition))
Concept	Course of the mental illness, care needs, utilization of care services and psychosocial effects (according to ICF)
Context	No specific context

### Exclusion criteria

- No Long COVID: reports that did not address the Long COVID Syndrome
- No mental health diagnosis: reports that did not include a mental health diagnosis, ensuring that we only considered research with specific mental disorders (e.g., depression, anxiety) and not based on, e.g., self-reported health
- No mental health diagnosis before Long COVID: reports in which mental health diagnoses were not made before the onset of Long COVID
- Construct not focusing on a mental health diagnosis: reports that focused on constructs unrelated to recognized mental health disorders (e.g., the course of Long COVID)
- Wrong publication type: reports that were not original research publications, such as conference abstracts, or commentaries
- No English or German language: reports not published in English or German to ensure we could clearly and accurately understand the research

### Information sources

We conducted the literature search in the following bibliographic databases: PubMed, Embase, and PsycINFO. Grey literature, preprints and unpublished reports were not considered.

### Search

We utilized the search terms contained in the titles and abstracts of relevant reports, as well as the index terms used to describe the reports, to develop a comprehensive search strategy for PubMed, Embase, and PsycINFO, following the PRESS guidelines [39] on November 1, 2023. The search strategy can be found in Appendix I.

In addition, we carried out a forward and backward citation tracking for all included publications on 20 June 2024 by using the webapp citation chaser (https://estech.shinyapps.io/citationchaser/). This is a transparent and efficient literature research method in which both subsequently cited publications (forward) and previously cited works (backward) are analyzed to identify relevant sources and connections in a particular field of research [40].

### Selection of sources of evidence

Following the literature search, we compiled all included records, uploaded them into the reference management software EndNote Version 2020.3 and removed duplicates. Six reviewers participated in all stages of the selection process (AN, AZ, CM, MG, SD, SM). During a pilot test (5% of the records), all six reviewers independently screened the titles and abstracts and assessed them against the inclusion criteria. For the literature screening, we used the web-based tool Rayyan [41]. Following the pilot phase, a group discussion was conducted with the six reviewers involved in the selection process to resolve any questions or conflicts.

Afterwards, the remaining 95% of the records were divided into three parts and distributed among the group of six reviewers, who worked in pairs. Each reviewer in the pair conducted the screening independently. For this purpose, three subgroups were created in Rayyan, with each pair working separately. After the independent screening, the pair met to reach a consensus. If any disagreements or uncertainties arose, the pairs consulted one of the other pairs and discussed the inconsistencies until they reached a consensus. After all pairs completed their screenings, a final meeting with all six reviewers was held to resolve any remaining questions and clarify any outstanding issues.

Afterwards, we organized the full texts of the relevant records. The full text screening was again conducted by the three pairs of reviewers. Within the three pairs, two independent reviewers screened the reports based on the inclusion criteria and documented the reasons for excluding reports that did not meet the inclusion criteria. Conflicts that arose between the reviewers during this phase of the selection process were discussed as previously described.

### Data charting process

Six reviewers (AN, AZ, CM, MG, SD, SM) independently extracted data from three reports with different study designs for pilot testing, as recommended [42]. Subsequently, all six reviewers in pairs extracted the data using a data extraction tool. Within each pair, both reviewers independently conducted data extraction and afterwards compared their results, resolving discrepancies through consensus. The data extraction tool can be found in Appendix II.

### Data items

The extracted data contains specific details about:

- Author(s)
- Year of publication
- Journal
- Country or countries reported on
- Healthcare settings (outpatient, inpatient)
- Aim
- Study design
- Methods of data collection
- Population (N, diagnosis, gender/sex as indicated by the authors, age, Long COVID)
- Impact of Long COVID on the defined population (outcome of the study) course of the mental illness, 2) need for care, 3) utilization of care services 4) psychosocial aspects (health status, physical functions/ structures of the person, their activities/ participation as well as environmental factors and personal factors) according to the ICF [36].

We performed a pilot test of the extraction tool, as recommended [38]. The pilot test was conducted on three reports, each featuring study designs that were as diverse as possible, with all six reviewers participating independently. We discussed the results until we reached consensus and, in cases of discrepancies, consulted a third person involved in the review.

### Synthesis of results

We aimed at providing an overview of the situation of individuals with mental health disorders and Long COVID. For presentation of results, we used the terms for Long COVID, as applied in the included studies. Study results were categorized according to the four predefined aspects of our review: 1) the course of the mental health disorder, 2) care needs, 3) utilization of care services and 4) psychosocial aspects. We reported on both quantitative and qualitative data and described similarities and differences within and between the core aspects to identify patterns and relevant themes.

## Results

### Selection of sources of evidence

**Fig. 1:**
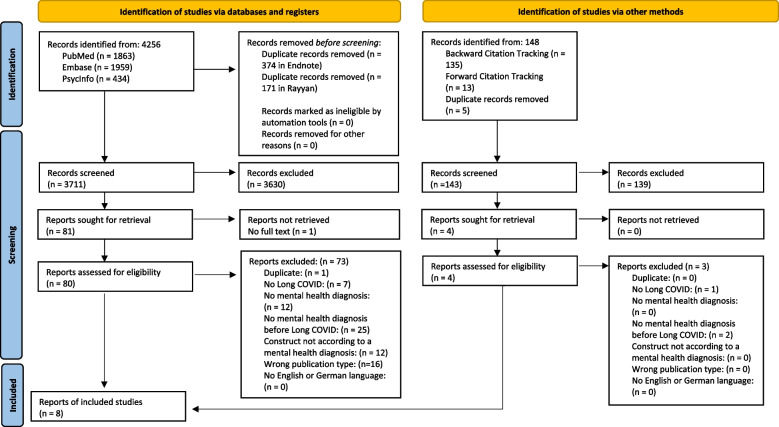
Flow chart according to PRISMA 2022

We identified 4256 records from all three databases searched. After removing duplicates, we screened the titles and abstracts of the remaining 3711 unique records and identified 81 potentially relevant reports, of which 80 were assessed for full-text eligibility. The one report we were unable to retrieve was a literature review published in 2022 on the topic of paediatric anxiety and depression during the COVID-19 pandemic [43]. After completing the full-text screening, we included seven reports in our scoping review. Through backward and forward citation tracking of these reports, we identified 143 additional records. After removing duplicates, four reports were deemed relevant. Only one of these reports met the inclusion criteria. Therefore, our scoping review includes a total of eight reports. An overview of the selection process is provided in the PRISMA 2020 Flow chart [44], which can be found in Fig. 1.

A reference list of included reports is provided in Appendix III, while the reference list of excluded reports, along with the reasons for their exclusion, is available in Appendix IV.

### Characteristics of sources of evidence

Half of the reports were published in 2022 (4/8), followed by 2023 (3/8), and one report in 2024 (1/8). The countries reported in the publications included the United States (5/8), with several publications also reporting on multiple countries: Italy, Japan, Austria, Belgium, United States and Canada (2/8). One publication did not specify a country, as it cited multiple studies (1/8). An overview can be found in Table 2.

**Table 2:** Included reports

**No**	**First author**	**Year**	**Journal**	**Country** (being reported on)
1	Chen [45]	2024	American Journal of Physical Medicine & Rehabilitation	United States
2	Efstathiou [46]	2022	Experimental and Therapeutic medicine	Not stated (citation of multiple studies)
3	Farooqi [47]	2022	Neuropsychiatric Disease and Treatment	United States
4	Ferrando [48]	2023	Frontiers in Psychiatry	United States
5	Hovagemyan [49]	2023	Frontiers in Psychiatry	Italy, Japan, Austria, Belgium, United States, Canada
6	Jyonouchi [50]	2022	Journal of Personalized Medicine	United States
7	Light [51]	2022	Case Reports in Neurology	Italy, Japan, Austria, Belgium, United States, Canada
8	Strawn [52]	2023	Journal of the American Academy of Child & Adolescent Psychiatry	United States

The majority of included studies investigated humans with a diagnosis of anxiety or depression and used quantitative methods or a combination of quantitative and qualitative methods. Two of the included studies were reviews: one scoping review [49] and one a review that can be specified as narrative [46]. One primary study [47] is included in the scoping review [49], which we also included in our scoping review. Among the six original studies, two were case reports [50, 51], two employed a retrospective design [45, 47], one utilized a prospective design [52], and one adopted a cross-sectional study design [48].

Three studies targeted children or adolescents [45, 50, 52] and three adults [47, 48, 51]. The age of participants was not specified in two of the studies [46, 49]. An overview of the study characteristics and methods is provided in Table 3.

**Table 3:** Study characteristics and methods

**No**	**First author**	**Population** N^a^ Disorders^b^ Sex/Gender %^c^ Age (mean)^d^ Syndrome (Post, Long COVID etc.)^e^	**Healthcare settings** (outpatient, inpatient)	**Aim**	**Study design**	**Methods**
1	Chen [45]	*N* = 93 Disorders: mood disorders (anxiety, depression, obsessive–compulsive disorder) Sex (female): 59 (63%) Age (years (SD)): 13.7 (4.3) Syndrome: Long COVID	Inpatient	“[…] to explore the influence of preexisting conditions and other potential predictive factors on symptoms and quality of life in a cohort of children with long COVID that presented to a paediatric long COVID clinic in Maryland.”	Single-center retrospective chart review	Quantitative: standardized intake surveys and physician documentation in medical records for self-reported symptoms; PedsQL inventories for assessment of quality of life
2	Efstathiou [46]	Primary study 1 [53]: *N* = 402 patients (COVID-19 survivors) Primary study 2 [54]: *N* = 226 patients (COVID-19 survivors) Primary study 3 [55]: *N* = 200 hospitalized patients with severe-to-critical COVID-19 infection Disorders: Primary study 1: pre-existing psychiatric diagnosis Primary study 2: pre-existing psychiatric diagnosis Primary study 3: pre-existing mental health issues Age: not reported Gender: not reported Syndrome: Long COVID	Not applicable	“[…] to broaden the understanding of neuropsychiatric sequelae in COVID-19 survivors and to develop targeted, evidence-based approaches for integrated patient care.”	(Narrative) Review Primary study 1: Cohort study Primary study 2: Cohort study Primary study 3: retrospective case series	Primary study 1: self-rated psychometric instruments Primary study 2: self-rated psychometric instruments Primary study 3: not reported
3	Farooqi [47]	*N* = 30 (past history of mental disorder(s) = 17) Disorders: depressive disorder, anxiety disorder, both depressive and anxiety disorder, adjustment disorder Gender (female): 26 (87%) (past history = 14 (82%)) Age (years (SD)): 51.3 (12.6) (past history = 55.9 (10.9)) Syndrome: Long-Term Sequelae of SARS-CoV2 Infection	Outpatient	“[…] to describe the sociodemographic, diagnostic and treatment characteristics of patients evaluated in an outpatient psychiatric setting for PASC.”	Retrospective review of medical records and consultation in outpatient setting	Quantitative: Patient Health Questionnaire-9 (PHQ-9), Generalized Anxiety Disorder Questionnaire (GAD-7), Fatigue Severity Scale (FSS), Columbia Suicide Severity Rating Scale (C-SSRS) for assessment of severity
4	Ferrando [48]	*N* = 75 Disorders: generalized anxiety disorder (GAD); Post-traumatic stress disorder (PTSD) Gender: 53 female, 22 male Age (years): 43.5 Syndrome: post-acute sequelae of COVID-19	Outpatient	“[…] to document the cross-sectional prevalence, characteristics and clinical correlates of anxiety and post-traumatic stress in a study of neuropsychiatric sequelae of COVID-19.”	Cross-sectional study	Qualitative: structured interviews Quantitative: Generalized Anxiety Disorder-7 (GAD-7) questionnaire, Post Traumatic Stress Disorder Checklist for DSM-5 (PCL-5); Neurocognitive assessments and collection of medical (e.g. medical history, COVID-19 disease), psychiatric (e.g. previous treatments, substance use) and socio-demographic data
5	Hovagemyan [49]	*N* = 9137 (11 studies), 1,502 (16.4%) of which were patients with a psychiatric history Disorders: adjustment disorder, alcohol abuse, anxiety, bipolar disorder, depression, eating disorder, obsessive–compulsive disorder, post-traumatic stress disorder, sleep disorder, psychiatric disease without specification Gender: not stated Age: not stated Syndrome: Long COVID	Outpatient & inpatient	“[…] to evaluate the present state of research of the possible effect of long COVID on the mental health of patients with prior psychiatric comorbidities or a previous history of psychiatric illness.”	Scoping review Primary studies: retrospective studies (*n* = 3), cross-sectional studies (*n* = 3), case reports (*n* = 2), cohort studies (*n* = 2), descriptive study (*n* = 1)	Not applicable
6	Jyonouchi [50]	*N* = 3 Disorder: Autism Spectrum disorder (ASD) Gender: 1 female, 2 male Age (years): Case 1: female = 15 Case 2: male = 25 Case 3: male = 9 Syndrome: Long COVID	Not specified	“[…] we present three ASD cases who presented with markedly worsening neuropsychiatric symptoms following COVID-19 exposure and subsequent difficulty in managing the post-COVID neuropsychiatric symptoms”	Case reports	Not applicable
7	Light [51]	*N* = 1 Disorders: premorbid attention-deficit hyperactivity disorder, mood difficulties, family history of vascular dementia, several somatic comorbidities Gender: female Age (years): 62 Syndrome: Long COVID	Outpatient & inpatient	“This case describes a complex presentation of a constellation of cognitive and emotive symptoms that may increasingly represent what neuropsychologists will encounter in the outpatient neuropsychology clinic setting in the coming months/years.”	Case report	Not applicable
8	Strawn [52]	*N* = 26 Disorders: generalized anxiety disorder, separation anxiety, social anxiety disorder, panic disorder, attention-deficit/hyperactivity disorder, history of depressive disorder Gender: 14.8% male Age (years): 14.3 Syndrome: Long COVID	Not specified	“We examined the longitudinal course of anxiety, including following laboratory-confirmed SARS-CoV- 2 infection in affected adolescents.” “[…] to examine differences in anxiety symptoms before and after SARS-CoV-2 infection”	Prospective longitudinal study	Qualitative: structured interviews Quantitative: Generalized Anxiety Disorder-7 (GAD-7), Quick Inventory of Depressive Symptomatology (QIDS), clinician-rated Clinical Global Impressions-Severity (CGI-S); Monte-Carlo Simulation

^a^number of patients or cases studied; ^b^disorder(s) which were considered in the study, only diagnosed disorders (not symptoms only) were considered; ^c^sex or gender used according to how the authors declared it in their manuscript; ^d^age of the study participants investigated; ^e^terminology derived from the respective reports (e.g., Post COVID, Long COVID, etc.)

### Results of individual sources

Even though we specifically examined the study results in four different categories, we predominantly found results describing the course of the mental health disorder (category a). Two studies [50, 51] also added information on the need for mental health care (category b). We could not find any results on utilization of health care services (category c) or psychosocial aspects (category d).

## Findings on the course of mental health

### Further mental health issues

TThe results of three studies show that for those suffering from Long COVID and having pre-existing mental health disorders, e.g. mood disorders or depressive symptoms, the risk of having further mental health or related issues, e.g. change in appetite, first-time-occurring anxiety or depression, dizziness/light-headedness symptoms and post-traumatic stress disorder (PTSD), increased [45, 46, 48].

The narrative review found non-significant associations for PTSD and Long COVID [46]. In a retrospective review of medical records and consultations in an outpatient setting, it was indicated that individuals with a past psychiatric history were not more likely to experience new symptoms of anxiety, depression, or insomnia after contracting COVID-19 compared to those without such a history [47].

### Worsening of mental health symptoms

The most commonly reported disorders were anxiety disorders, mentioned in five reports [45, 47–49, 52], as well as depression, cited in four reports [45, 47, 49, 52]. Mood disorders were reported in two studies [45, 51]. One report focused on Autism Spectrum Disorder (ASD) [50]. Four reports indicate an increased prevalence of worsening symptoms in individuals with pre-existing mental health diagnoses [45, 46, 48, 49]. According to Efstathiou et al., deterioration was shown both during hospitalization due to COVID-19 and after discharge [46]. Strawn et al. further reported that anxiety symptoms, clinical global assessment of severity and depressive symptoms increase significantly after SARS-CoV-2 infection in adolescents with pre-existing anxiety disorders [52]. One of the eight included reports [47] did not report respective associations.

### Persisting mental health symptoms

In one report, it was stated that people with a history of mental health issues may be at a higher risk of experiencing persistent symptoms of depression [46]. Another report found a continued experience of cognitive and emotive difficulties despite ongoing treatments (psychopharmacotherapy, psychotherapy, speech-language pathology) [51].

### Findings on the need for mental health care

One study illustrated the profound effects of COVID-19 on neuropsychiatric symptoms in subjects with autism spectrum disorders and the difficulty of managing Long COVID symptoms [50]. Further, another report underlined the need for careful use of differential diagnosis in COVID-19 patients with multiple risk factors for neuropsychological and psychiatric disorders that make them more susceptible to long-term neurological complications post COVID-19 [51]. An overview of the results found can be found in Table 4.

**Table 4:** Studies included—results

No	First author	Category^a^	Results (quotes from the authors of the included reports or studies)
Studies with children and adolescents			
1	Chen [45]	a	“Children with preexisting mood disorders had a 2.7 greater odds of change-in-appetite symptoms (95% CI, 1.02–7.6; *P* = 0.04); patients with preexisting anxiety had a 5.5 greater odds of reporting anxiety symptoms during their initial long COVID visit (95% CI, 1.44–31.1; *P* = 0.01), and patients with preexisting depression had a 4.8 greater odds of reporting depression symptoms at their initial long COVID visit (95% CI, 1.08–29.4; *P* = 0.04). Interestingly, patients with preexisting mood disorders had a 6.1 greater odds of having dizziness/light-headedness symptoms (95% CI, 1.3–57.4; *P* = 0.02)”
		a	Pre-existing mood disorders were associated with a higher prevalence of worsening mental health symptoms (anxiety, *p* = 0.01; depression, *p* = 0.04), dizziness/light-headedness/vertigo (*p* = 0.02) and change in appetite (*p* = (*p* = 0.04)
6	Jyonouchi [50]	a	Case 1: brain fog, worsening fluctuation of ASD behavioral symptoms Case 2: neuropsychiatric symptoms, marked fatigue, loss of appetite and weight loss (…), worsened neuropsychiatric symptoms manifesting as severe mood swings and temper tantrums Case 3: acute exacerbation of neuropsychiatric symptoms apparently triggered by viral infection
		b	„The presented cases illustrate the profound effects of COVID-19 on neuropsychiatric symptoms in ASD subjects and the difficulty of managing long-COVID symptoms.”
8	Strawn [52]	a	Anxiety symptoms (measured with GAD-7), clinical global assessment of severity (CGI-S) and depressive symptoms (QIDS; apart from"hypersomnia") increase significantly after SARS-CoV-2 infection in adolescents with pre-existing anxiety disorders
Studies with adults			
3	Farooqi [47]	a	“[…] those with past psychiatric history were not more likely to report new symptoms of anxiety, depression, or insomnia following COVID-19 illness, than those with no past psychiatric history.”
4	Ferrando [48]	a	“Prior history of psychiatric treatment is also a strong predictor of anxiety and/or PTSD post-COVID-19, and likely constitutes a vulnerability for worsened symptoms.”
7	Light [51]	a	“[…] persistence of cognitive-emotive symptoms 8 months post hospital discharge; worsening attention, poor short-term memory, low average delayed memory, reduced executive functioning capacity, significant daytime fatigue […]”
		b	Currently independent in terms of basic activities of daily living functions, requires help to accomplish most instrumental functions
		c	Outpatient cognitive rehabilitation (speech and language pathologist, physical therapy)
Age not stated			
2	Efstathiou [46]	a	“Moreover, females, patients with a pre‑existing psychiatric diagnosis and patients who were managed at home exhibited increased levels in the majority of psychopathological measurements […].”
		a	“[…] patients with pre‑existing mental health issues presented a deterioration of their symptoms both during hospitalization and after discharge […].”
		a	“As regards the impact of comorbidities, while associations with either depression […] or both anxiety and depression symptoms […], have been suggested, other studies have not found any association between comorbidities and ‘long COVID’ symptoms […].”
		a	“There is evidence to indicate that patients with a history of mental health issues may be at a higher risk of presenting persisting depression symptoms […]”
		a	“Crucially, patients with pre‑existing mental health problems are likely to be at a greater risk of presenting PTSD […], even when controlling for other demographic or clinically relevant factors […]; nevertheless, non‑significant associations have also been reported […].”
5	Hovagemyan [49]	a	11 studies were included
			„6/11 studies found an effect of Long-COVID (…) with either a worsening in length or severity “„4/11 studies did not find any correlations between worsening symptoms and psychiatric history.“„It seems that studies with a self-report system tend to show outcomes for PWPH that are worse than for patients without prior psychiatric morbidities (42.9%) than studies with a medical record retrieval system (0%).“

^a^ category: a = course of disease (mental health); b = need for care (mental health); c = utilization of health care services (mental health); d = psychosocial aspects (mental health); e = other

*Abbreviations*: *ASD* Autism Spectrum Disorder; *PTSD* Post-Traumatic Stress Disorder

## Discussion

### Summary of evidence

In this scoping review we identified eight studies examining Long COVID in people with pre-existing mental health disorders. The majority of studies focused on the course of mental health disorders in individuals with pre-existing conditions experiencing Long COVID. On the one hand, there are indications that individuals with pre-existing mental health diagnoses, such as mood disorders, anxiety, and depression, are more likely to experience a worsening of their symptoms in the context of Long COVID, both during hospitalization and after discharge [46]. People with mood disorders reported an increased risk of additional symptoms, including changes in appetite, dizziness, anxiety, and PTSD [45, 46, 48]. On the other hand, there is evidence showing that individuals with a history of psychiatric disorders may be more likely to experience new symptoms of anxiety, depression, or insomnia following a COVID-19 infection [47]. Only two studies [50, 51] mentioned the need for mental health care. One study highlighted the impact of COVID-19 on neuropsychiatric symptoms in individuals with ASD [50], while another stressed the need for careful differential diagnosis in patients at higher risk for long-term neurological complications [51]. However, there were no findings regarding the utilization of mental health care services or psychosocial aspects.

This inconsistency has also been noted in other studies and is in line with the scoping review by Hovagemyan et al., indicating that while 6 out of 11 studies showed an effect of Long COVID on symptom worsening for patients with psychiatric histories, 4 studies did not find any correlation between worsening symptoms and psychiatric history [49].

This raises the question of which factors may have contributed to the inconsistency in the findings. First, the geographical context may play a role, as the perception of mental health varies across different cultures and regions [56]. Differences in the quality of healthcare, access to resources, or the availability of social support networks could possibly affect how symptoms develop and are managed.

Second, another important factor could have been the social isolation resulting from the confinement measures and contact restrictions imposed during the pandemic, which varied across countries [57]. It is possible that the mental health of individuals who experienced greater isolation or had limited social interactions due to these restrictions were more prone to deterioration. Third, another factor could be the severity of the Long COVID illness itself, as severe acute COVID-19 has been linked to long-term mental health issues among individuals recovering in the general population [58]. However, the included studies did not provide conclusive evidence regarding the impact of these factors.

Notably, healthcare utilization and psychosocial impacts have been largely underexplored in the research on Long COVID in individuals with pre-existing mental health disorders. This gap may reflect a broader lack of research on the intersection between infectious diseases and mental health conditions. However, if the intersection is investigated, it has not yet been approached from a biopsychosocial perspective according to the ICF. This highlights the need for future studies to address both healthcare utilization patterns and psychosocial outcomes.

### Implications for clinical practice

First, given the increased risk of mental health deterioration in people with pre-existing mental health diagnoses, especially tertiary preventive measures regarding a further complication of mental health problems, e.g., therapeutic approaches, should be considered. While a systematic review [59] and a scoping review [60] identified a variety of therapeutic approaches targeting cognitive and mental health outcomes in populations with Long COVID, evidence specifically for populations with pre-existing mental health problems is currently lacking. Early identification of at-risk populations and the implementation of preventive strategies could possibly mitigate the severity of Long COVID-related mental health issues. For example, incorporating routine mental health screenings in COVID-19 survivors, especially those with known psychiatric histories, could help to prevent worsening symptoms or relapses.

Second, one of the major challenges is differentiating between SARS-CoV-2-induced somatic and psychological disorders, exacerbations of pre-existing conditions, and pandemic-related psychosocial stressors. The interaction between these factors can lead to mutual reinforcement, complicating the clinical picture and making it difficult to pinpoint the exact causes of neuropsychiatric symptoms [2]. This observation remains valid, which underscores the need to pay greater attention to patients with both pre-existing mental health disorders and Long COVID.

### Implications for research

First, the current literature on mental health sequelae following a COVID-19 infection reveals a noticeable limitation: many studies exclude participants with a psychiatric history [46]. This limits the generalizability of findings and potentially overlooks a high-risk group that could provide valuable insights into the interplay between pre-existing conditions and COVID-19 outcomes. Hovagemyan et al. [49] echo this concern, noting that the majority of studies on mental health outcomes in COVID-19 exclude individuals with prior psychiatric histories, focusing instead on new-onset neuropsychiatric outcomes. This exclusion neglects those with pre-existing psychiatric conditions, despite their higher risk for developing new symptoms, symptom persistence or experiencing relapses. By excluding these participants, many studies limit the generalizability of their findings and fail to account for a high-risk group. Accordingly, Ferrando et al. [48] argue that prior psychiatric history should always be included in clinical assessments to stratify risk for developing neuropsychiatric symptoms post-COVID-19. A more inclusive approach that incorporates this group would allow for a more nuanced and meaningful analysis.

Second, self-report systems tend to report worse outcomes for patients with pre-existing psychiatric histories compared to studies utilizing medical record retrieval systems. Specifically, 42.9% of self-report studies noted poorer outcomes for those with a psychiatric history, compared to 0% in studies relying on medical records [49]. This discrepancy underscores the need for standardized and objective methods of assessment to better understand the true impact of COVID-19 on this population.

Third, we found mainly results describing the course of mental illness (e.g. progression of symptoms, persistence and further mental health problems) and, secondarily, the need for mental health care. However, research gaps related to the utilization of health care services and the psychosocial aspects of mental health in people with prior mental illness and Long COVID could be identified. There are limited data on how these individuals access mental health care services. This gap is noteworthy because understanding service utilization patterns is critical for designing effective mental health interventions and ensuring that care actually reaches those in need. Without this information, it is difficult to determine whether existing healthcare systems are adequately responding to the demand for mental health services. There is also a lack of data on the psychosocial dimensions of mental health in people with prior mental illness and Long COVID, such as the impact on relationships, social functioning, and overall quality of life. Lastly, while there is evidence for the effectiveness of several types of interventions (e.g., cognitive-behavioral therapy and acceptance and commitment therapy) for mental health outcomes in people with Long COVID, respective studies describing effectiveness specifically in individuals with pre-existing mental health problems are urgently needed [59, 60]. These aspects are essential for a holistic understanding of mental health, as they influence not only the individual’s wellbeing but also their capacity to recover and reintegrate into daily life.

In addition, it is crucial to consolidate existing research findings, for example, to inform policy decisions. Initial initiatives, such as the Long COVID Research Networks funded by the German Federal Ministry of Health, have already been established to develop and disseminate scientific knowledge on the treatment of Long COVID, including ME/CFS. This involves one network dedicated to adults and another specializing in the care of children and adolescents. The goal of these networks is to ensure that research findings will be translated into clinical practice. At the same time, data from routine healthcare will be made available to researchers, fostering a continuous and reciprocal exchange between science and practice [61].

Future research on Long COVID in individuals with pre-existing mental health disorders should also integrate healthcare utilization and psychosocial aspects. Longitudinal studies would be valuable in examining the long-term effects of Long COVID on both physical and mental health outcomes. Additionally, the use of standardized instruments to assess healthcare utilization and psychosocial impacts is essential for ensuring consistency and comprehensiveness across studies. Furthermore, mixed-methods approaches, combining both qualitative and quantitative data, would provide a more nuanced understanding of the healthcare needs and psychosocial consequences of this population.

### Strengths and limitations

One of the strengths is that we closely adhered to the recommendations of the Joanna Briggs Institute (JBI) for conducting scoping reviews. Furthermore, both the screening and data extraction processes were conducted independently by two reviewers, ensuring a higher level of reliability and reducing the risk of bias in the selection and analysis of the studies. By focusing on individuals with pre-existing mental health problems, we address a population of high interest for researchers and clinicians alike, pointing out promising areas for further study.

Despite its strengths, this scoping review has some limitations. First, the search period was limited and may no longer fully capture the most recent studies in this rapidly evolving field. Second, we only used three databases for our literature search and did not include a search of grey literature, which means there is a possibility that we may have missed some relevant studies. However, by applying a thorough search strategy and use of forward and backward citation tracking, we aimed to reduce bias due to potentially missed studies.

Third, despite the use of forward citation tracking, relevant studies published after November 2023 may not have been included in the analysis. In the future, an update of this scoping review could be necessary to incorporate newer relevant studies.

## Conclusion

This scoping review provides indications that people with prior mental illness and Long COVID may represent a risk group for mental health deterioration, symptom persistence and the development of additional symptoms in the context of Long COVID. Yet, the exclusion of patients with psychiatric histories from many studies limits our understanding of Long COVID’s full impact on mental health. Future research should aim to include this vulnerable population, use standardized assessment methods, and explore broader aspects such as health care utilization and psychosocial factors. Development and evaluation of preventive and early intervention strategies, tailored to those with pre-existing conditions, are essential to mitigate the long-term mental health effects of COVID-19.

## Supplementary Information

Supplementary Material 1

## Data Availability

The materials and data can be obtained from the authors on request.
