# Supplementary material for: Long COVID in people with mental health disorders: a scoping review
Source: BMC Psychiatry. 2025 Jul 1;25:669. doi: 10.1186/s12888-025-06935-9 (PMC12219037; doi:10.1186/s12888-025-06935-9)
Supplement: Supplementary file 1 — Supplementary Material 1 [file 12888_2025_6935_MOESM1_ESM.docx]

# **Appendices**

### **Appendix I: Search strategy**

**Embase**

Search conducted *on November 1, 2023.*

(mental disease.mp. or exp mental disease/ or (exp behavior/ or behavior.mp.) or 'Alzheimer disease'.tw. or 'dement*'.tw. or 'delirium'.tw. or 'Alzheimer*'.tw. or 'substance abuse'.tw. or 'alcohol drinking'.tw. or 'schizophrenia'.tw. or 'schizotype'.tw. or 'delusional disorders'.tw. or 'psychotic'.tw. or 'schizoaffective'.tw. or 'affective disorders.tw. or 'manic disorder*'.tw. or 'bipolar'.tw. or 'depressive'.tw. or 'depression'.tw. or 'somatoform disorders'.tw. or 'phobic disorder*'.tw. or 'anxiety'.tw. or 'compulsive disorder*'.tw. or ('reaction to severe stress.mp. and adjustment disorder*'.tw.) or 'dissociative disorder*'.tw. or 'neurotic disorder*'.tw. or 'obsessive-compulsive disorder'.tw. or 'eating disorder*'.tw. or 'sleeping disorder*'.tw. or 'personality disorder*'.tw. or 'intelligence reduction'.tw. or 'developmental disorder*'.tw. or 'hyperkinetic disorder*'.tw. or 'disorder* of social behavi*'.tw. or 'emotional disorder*'.tw. or 'social function disorder*'.tw. or 'tic disorder*'.tw. or 'neural disorder*'.tw. or 'mental development disorder*'.tw. or 'trauma disorder*'.tw. or 'stress-related disorder*'.tw. or 'somatic stress disorder*'.tw. or 'elimination disorder*'.tw. or 'sleep-wake disorder*'.tw. or 'gender dysphoria'.tw. or 'disruptive disorder*'.tw. or 'impulse control disorder*'.tw. or 'social behavi* disorder*'.tw. or 'psychotropic substance*'.tw. or 'dependent behavi*'.tw. or 'neurocognitive disorder*'.tw. or 'paraphilic disorder*'.tw. or 'drug-induced movement disorder*'.tw.) and ('COVID-19 sequela*' or (('COVID-19' or 'Sars-CoV-2' or '2019 Novel Coronavirus' or '2019-nCoV' or 'Coronavirus Disease 2019' or 'Coronavirus Disease-19' or 'SARS Coronavirus 2' or 'Severe Acute Respiratory Syndrome Coronavirus 2') and 'sequela*') or 'post acute sequelae of Sars-CoV-2' or ('PASC' and ('COVID-19' or 'Sars-CoV-2' or '2019 Novel Coronavirus' or '2019-nCoV' or 'Coronavirus Disease 2019' or 'Coronavirus Disease-19' or 'SARS Coronavirus 2' or 'Severe Acute Respiratory Syndrome Coronavirus 2')) or 'post acute sequelae of COVID' or (('post-intensive care syndrome' or 'postintensive care syndrome') and ('COVID-19' or 'Sars-CoV-2' or '2019 Novel Coronavirus' or '2019-nCoV' or 'Coronavirus Disease 2019' or 'Coronavirus Disease-19' or 'SARS Coronavirus 2' or 'Severe Acute Respiratory Syndrome Coronavirus 2')) or 'post COVID condition*' or ('PCC' and ('COVID-19' or 'Sars-CoV-2' or '2019 Novel Coronavirus' or '2019-nCoV' or 'Coronavirus Disease 2019' or 'Coronavirus Disease-19' or 'SARS Coronavirus 2' or 'Severe Acute Respiratory Syndrome Coronavirus 2')) or 'convalescent COVID-19' or 'long haul COVID' or 'COVID long haul*' or 'long COVID' or 'long term COVID' or 'COVID-19 survivor*' or 'post COVID-19 symptom*' or 'chronic COVID syndrome' or 'post COVID syndrome' or 'post COVID-19 neurological syndrome' or 'post acute COVID-19' or 'post-acute COVID-19 syndrome' or 'COVID-19 post-intensive care syndrome').tw.

Hits: 1959

**PsycInfo**

((MA mental disorders OR MA (behavior and behavior mechanisms ) OR AB dementia OR AB alzheimer's disease OR AB dement* OR AB delirium OR AB alzheimers OR AB substance abuse OR AB alcohol drinking OR AB schizophrenia OR AB schizotype OR AB delusional disorder ) OR ( AB psychotic OR AB schizoaffective OR AB affective disorder OR AB manic disorder* OR AB bipolar OR AB depressive OR AB depression OR AB somatoform disorders OR AB phobic disorder* OR AB anxiety OR AB compulsive disorder* OR AB ( reaction to severe stress and adjustment disorder*) ) OR AB dissociative disorder* OR AB neurotic disorder* OR AB obsessive compulsive disorder* OR AB eating disorder* OR AB sleeping disorder* OR AB personality disorder* OR AB intelligence reduction OR AB developmental disorder* OR AB hyperkinetic disorder* OR AB disorder* of social behavi* OR AB emotional disorder* OR AB social function disorder* OR AB tic disorder* OR AB neural disorder* OR AB mental development disorder* OR AB trauma disorder* OR AB stress-related disorder* OR AB somatic stress disorder* OR AB elimination disorder* OR AB sleep-wake disorder* OR AB gender dysphoria OR AB disruptive disorder* OR AB impulse control disorder* OR AB social behavi* disorder* OR AB psychotropic substance OR AB dependent behavi* OR AB neurocognitive disorder* OR AB paraphilic disorder* OR AB drug-induced movement disorder*)) AND COVID-19 sequela*" OR (("COVID-19" OR "Sars-CoV-2" OR "2019 Novel Coronavirus" OR "2019-nCoV" OR "Coronavirus Disease 2019" OR "Coronavirus Disease-19" OR "SARS Coronavirus 2" OR "Severe Acute Respiratory Syndrome Coronavirus 2") AND sequela*) OR "post acute sequelae of Sars-CoV-2" OR ("PASC") AND ("COVID-19" OR "Sars-CoV-2" OR "2019 Novel Coronavirus" OR "2019-nCoV" OR "Coronavirus Disease 2019" OR "Coronavirus Disease-19" OR "SARS Coronavirus 2" OR "Severe Acute Respiratory Syndrome Coronavirus) AND sequela*) OR "post acute sequelae of COVID" OR (("post-intensive care syndrome" OR "postintensive care syndrome") AND ("COVID-19" OR "Sars-CoV-2" OR "2019 Novel Coronavirus" OR "2019-nCoV" OR "Coronavirus Disease 2019" OR "Coronavirus Disease-19" OR "SARS Coronavirus 2" OR "Severe Acute Respiratory Syndrome Coronavirus 2")) OR "post COVID condition*" OR ("PCC" AND ("COVID-19" OR "Sars-CoV-2" OR "2019 Novel Coronavirus" OR "2019-nCoV" OR "Coronavirus Disease 2019" OR "Coronavirus Disease-19" OR "SARS Coronavirus 2" OR "Severe Acute Respiratory Syndrome Coronavirus 2")) OR "convalescent COVID-19" OR "long haul COVID" OR "COVID long haul*" OR "long COVID" OR "long term COVID" OR "COVID-19 survivor*" OR "post COVID-19 symptom*" OR "chronic COVID syndrome" OR "post COVID syndrome" OR "post COVID-19 neurological syndrome" OR "post acute COVID-19" OR "post-acute COVID-19 syndrome" OR "COVID-19 post-intensive care syndrome"

Hits: 434

**PubMed**

(("mental disorders"[MeSH Terms]) OR ("Behavior and Behavior Mechanisms"[MeSH Terms]) OR

("dementia"[Title/Abstract]) OR (“Alzheimer disease” [Title/Abstract]) OR (“dement*” [Title/Abstract]) OR (“delirium” [Title/Abstract]) OR (“Alzheimer*” [Title/Abstract]) OR ("substance abuse"[Title/Abstract]) OR (“alcohol drinking” [Title/Abstract]) OR ("schizophrenia"[Title/Abstract]) OR (“schizotype” [Title/Abstract]) OR (“delusional disorders” [Title/Abstract]) OR (“psychotic” [Title/Abstract]) OR (“schizoaffective” [Title/Abstract]) OR ("affective disorders"[Title/Abstract]) OR (“manic disorder*” [Title/Abstract]) OR (“bipolar” [Title/Abstract]) OR (“depressive” [Title/Abstract]) OR ("depression"[Title/Abstract]) OR ("somatoform disorders"[Title/Abstract]) OR (“phobic disorder*” [Title/Abstract]) OR ("anxiety"[Title/Abstract]) OR (“compulsive disorder*” [Title/Abstract]) OR (“reaction to severe stress and adjustment disorder*” [Title/Abstract]) OR (“dissociative disorder*” [Title/Abstract]) OR (“neurotic disorder*” [Title/Abstract]) OR ("obsessive-compulsive disorder"[Title/Abstract]) OR ("eating disorder*"[Title/Abstract]) OR (“sleeping disorder*” [Title/Abstract]) OR ("personality disorder*"[Title/Abstract]) OR (“intelligence reduction” [Title/Abstract]) OR ("developmental disorder*"[Title/Abstract]) OR (“hyperkinetic disorder*” [Title/Abstract]) OR (“disorder* of social behavi* [Title/Abstract]) OR (“emotional disorder*” [Title/Abstract]) OR (“social function disorder* [Title/Abstract]) OR (“tic disorder*” [Title/Abstract]) OR (“neural disorder*” [Title/Abstract]) OR (“mental development disorder*” [Title/Abstract]) OR

(“trauma disorder*” [Title/Abstract]) OR (“stress-related disorder*” [Title/Abstract]) OR (“somatic stress disorder*” [Title/Abstract]) OR (“elimination disorder*” [Title/Abstract]) OR (“sleep-wake disorder*” [Title/Abstract]) OR (“gender dysphoria” [Title/Abstract]) OR (“disruptive disorder*” [Title/Abstract]) OR (“impulse control disorder*” [Title/Abstract]) OR (“social behavi* disorder*” [Title/Abstract]) OR (“psychotropic substance*” [Title/Abstract]) OR (“dependent behavi*” [Title/Abstract]) OR (“neurocognitive disorder*” [Title/Abstract]) OR (“paraphilic disorder*” [Title/Abstract]) OR (“drug-induced movement disorder*” [Title/Abstract])) AND ("COVID-19 sequela*" OR (("COVID-19" OR "Sars-CoV-2" OR "2019 Novel Coronavirus" OR "2019-nCoV" OR "Coronavirus Disease 2019" OR "Coronavirus Disease-19" OR "SARS Coronavirus 2" OR "Severe Acute Respiratory Syndrome Coronavirus 2") AND sequela*) OR "post acute sequelae of Sars-CoV-2" OR ("PASC" AND ("COVID-19" OR "Sars-CoV-2" OR "2019 Novel Coronavirus" OR "2019-nCoV" OR "Coronavirus Disease 2019" OR "Coronavirus Disease-19" OR "SARS Coronavirus 2" OR "Severe Acute Respiratory Syndrome Coronavirus 2")) OR "post acute sequelae of COVID" OR (("post-intensive care syndrome" OR "postintensive care syndrome") AND ("COVID-19" OR "Sars-CoV-2" OR "2019 Novel Coronavirus" OR "2019-nCoV" OR "Coronavirus Disease 2019" OR "Coronavirus Disease-19" OR "SARS Coronavirus 2" OR "Severe Acute Respiratory Syndrome Coronavirus 2")) OR "post COVID condition*" OR ("PCC" AND ("COVID-19" OR "Sars-CoV-2" OR "2019 Novel Coronavirus" OR "2019-nCoV" OR "Coronavirus Disease 2019" OR "Coronavirus Disease-19" OR "SARS Coronavirus 2" OR "Severe Acute Respiratory Syndrome Coronavirus 2")) OR "convalescent COVID-19" OR "long haul COVID" OR "COVID long haul*" OR "long COVID" OR "long term COVID" OR "COVID-19 survivor*" OR "post COVID-19 symptom*" OR "chronic COVID syndrome" OR "post COVID syndrome" OR "post COVID-19 neurological syndrome" OR "post acute COVID-19" OR "post-acute COVID-19 syndrome"[Supplementary Concept] OR "COVID-19 post-intensive care syndrome"[Supplementary Concept])

Hits: 1863

### **Appendix II: Data extraction instrument**

| Author |  |
| --- | --- |
| Year |  |
| Journal |  |
| Country in which the study was conducted |  |
| Methods of data extraction |  |
| Type of evidence source |  |
| Population (ICD-10 code if possible) |  |
| Impact of Long COVID on the defined population |  |
| Core aspect (1.) Course of disorder (mental health), 2.) Need for care, 3.) Utilization of care services 4.) Psychosocial aspects) |  |

**Appendix III: Included reports**

1. Chen EY, Morrow AK, Malone LA. Exploring the Influence of Pre-Existing Conditions and Infection Factors on Pediatric Long COVID Symptoms and Quality of Life. Am J Phys Med Rehabil. 2023.

2. Efstathiou V, Stefanou M-I, Demetriou M, Siafakas N, Makris M, Tsivgoulis G, et al. Long COVID and neuropsychiatric manifestations (Review). Experimental and therapeutic medicine. 2022;23(5):363-NA.

3. Farooqi M, Khan A, Jacobs A, D'Souza V, Consiglio F, Karmen CL, et al. Examining the Long-Term Sequelae of SARS-CoV2 Infection in Patients Seen in an Outpatient Psychiatric Department. Neuropsychiatric Disease and Treatment. 2022;18:1259-68.

4. Ferr, o SJ, Lynch S, Ferr, o N, Dornbush R, et al. Anxiety and posttraumatic stress in post-acute sequelae of COVID-19: prevalence, characteristics, comorbidity, and clinical correlates. Frontiers in Psychiatry. 2023;14:1160852.

5. Hovagemyan F, Dugerdil A, Braggion A, Mallet L, Flahault A. Psychiatric consequences and issues of long COVID on patients with prior psychiatric comorbidities: a scoping review. Frontiers in Psychiatry. 2023;14:1181767.

6. Jyonouchi H, Geng L, Rossignol DA, Frye RE. Long COVID Syndrome Presenting as Neuropsychiatric Exacerbations in Autism Spectrum Disorder: Insights for Treatment. Journal of Personalized Medicine. 2022;12(11):1815.

7. Light SN. The Combined Use of Neuropsychiatric and Neuropsychological Assessment Tools to Make a Differential Dementia Diagnosis in the Presence of "Long-Haul" COVID-19. Case Reports in Neurology. 2022;14(1):130-48.

8. Strawn JR, Mills JA, Schroeder HK, Neptune ZA, Specht A, Keeshin SW. The Impact of COVID-19 Infection and Characterization of Long COVID in Adolescents With Anxiety Disorders: A Prospective Longitudinal Study. Journal of the American Academy of Child & Adolescent Psychiatry. 2023;62(7):707-9.

**Appendix IV: Excluded reports**

1. Ahmed S, Hyman S, Kenney R, Fung S, Seixas A, Jean-Louis G, et al. Dysautonomia symptoms and quality of life in non-hospitalized neurology outpatients with post-acute sequelae of COVID-19. Neurology. 2023;100(17).

*Reason for exclusion:* Wrong publication type

2. Alghamdi SA, Alfares MA, Alsulami RA, Alghamdi AF, Almalawi AM, Alghamdi MS, et al. Post-COVID-19 Syndrome: Incidence, Risk Factor, and the Most Common Persisting Symptoms. Cureus. 2022;14(11):e32058.

*Reason for exclusion:* Construct not according to a mental health diagnosis

3. Alves EV, Beber BC. Self-perception of cognitive sequels in post-COVID-19 individuals. Dement Neuropsychol. 2023;17:e20220080.

*Reason for exclusion:* No Long Covid

4. Ambaliya C, Gupta D, Ankur A. Stress and Coping in Post Covid Sequelae in Indian Patients. Indian Journal of Public Health Research and Development. 2023;14(3):75-9.

*Reason for exclusion:* No mental health diagnosis before Long Covid

5. Aziz R, Brode WM, Kelley MA, Alvarado NS, Melamed E. CHARACTERISTICS OF LONG COVID PATIENTS PRESENTING TO A DEDICATED POST- COVID-19 CLINIC. Journal of General Internal Medicine. 2023;38:S108.

*Reason for exclusion:* No mental health diagnosis

6. Barh D, Tiwari S, Andrade BS, Weener ME, Góes-Neto A, Azevedo V, et al. A novel multi-omics-based highly accurate prediction of symptoms, comorbid conditions, and possible long-term complications of COVID-19. Mol Omics. 2021;17(2):317-37.

*Reason for exclusion:* No mental health diagnosis before Long Covid

7. Baslet G, Aybek S, Ducharme S, Modirrousta M, ana, Nicholson TR. Neuropsychiatry's Role in the Postacute Sequelae of COVID-19: Report From the American Neuropsychiatric Association Committee on Research. The Journal of Neuropsychiatry and Clinical Neurosciences. 2022;34(4):341-50.

*Reason for exclusion:* No mental health diagnosis before Long Covid

8. Baumeister A, Göritz AS, Benoy C, Jelinek L, Moritz S. Long-COVID or long before? Neurocognitive deficits in people with COVID-19. Psychiatry Res. 2022;317:114822.

*Reason for exclusion:* No mental health diagnosis

9. Beatty MC, Verma AK. Clinical Time Course of Post-Acute Sequelae of SARS-CoV-2 Cardiovascular Syndrome. Circulation. 2022;146.

*Reason for exclusion:* Wrong publication type

10. Beharry M. Pediatric Anxiety and Depression in the Time of COVID-19. Pediatr Ann. 2022;51(4):e154-e60.

*Reason for exclusion:* No Fulltext

11. Benoit-Piau J, Tremblay K, Piche A, Dallaire F, Belanger M, d'Entremont MA, et al. Long-Term Consequences of COVID-19 in Predominantly Immunonaive Patients: A Canadian Prospective Population-Based Study. Journal of Clinical Medicine. 2023;12(18):5939.

*Reason for exclusion:* No mental health diagnosis

12. Bilu Y, Flaks-Manov N, Goldshtein I, Bivas-Benita M, Akiva P, Bodenheimer G, et al. Youth Mental Health Outcomes up to Two Years After SARS-CoV-2 Infection Long-COVID or Long-Pandemic Syndrome: A Retrospective Cohort Study. Journal of Adolescent Health. 2023;73(4):701-6.

*Reason for exclusion:* Construct not according to a mental health diagnosis

13. Byrne A, Barber R, Lim CH. Impact of the COVID-19 pandemic - a mental health service perspective. Progress in Neurology and Psychiatry. 2021;25(2):27-33b.

*Reason for exclusion:* No Long COVID

14. Chaban O, Khaustova O, Assonov D. P.0370 Escitalopram efficacy in post-covid depression treatment: a pilot study. European Neuropsychopharmacology. 2021;53:S270.

*Reason for exclusion:* Wrong publication type

15. Chang J, Kim CH. Managing perceived distress to COVID-19 infection on post-COVID-19 condition. Early Intervention in Psychiatry. 2023;17:317.

*Reason for exclusion:* Wrong publication type

16. Damant RW, Rourke L, Cui Y, Lam GY, Smith MP, Fuhr DP, et al. Reliability and validity of the post COVID-19 condition stigma questionnaire: A prospective cohort study. eClinicalMedicine. 2023;55:101755.

*Reason for exclusion:* No mental health diagnosis

17. Davis PB, Wang Q, Xu R. Reply to "Post-COVID 19 neurological syndrome: A new risk factor that modifies the prognosis of patients with dementia". Alzheimer's & Dementia: The Journal of the Alzheimer's Association. 2022;18(3):544.

*Reason for exclusion:* Wrong publication type

18. Delgado-Alonso C, Cuevas C, Oliver-Mas S, Díez-Cirarda M, Delgado-Álvarez A, Gil-Moreno MJ, et al. Fatigue and Cognitive Dysfunction Are Associated with Occupational Status in Post-COVID Syndrome. Int J Environ Res Public Health. 2022;19(20).

*Reason for exclusion:* No mental health diagnosis

19. Durstenfeld MS, Peluso MJ, Peyser ND, Lin F, Knight SJ, Djibo A, et al. Factors Associated with Long Covid Symptoms in an Online Cohort Study. medRxiv. 2022.

*Reason for exclusion:* Wrong publication type

20. Durstenfeld MS, Peluso MJ, Peyser ND, Lin F, Knight SJ, Djibo A, et al. Factors Associated with Long COVID Symptoms in an Online Cohort Study. Open Forum Infectious Diseases. 2023;10(2):ofad047.

*Reason for exclusion:* No mental health diagnosis

21. Ebbesen BD, Giordano R, Valera-Calero JA, Hedegaard JN, Fernández-de-las-Peñas C, Arendt-Nielsen L. Prevalence and Risk Factors of De Novo Widespread Post-COVID Pain in Nonhospitalized COVID-19 Survivors: A Nationwide Exploratory Population-Based Survey. The Journal of Pain. 2023.

*Reason for exclusion:* Construct not according to a mental health diagnosis

22. Efstathiou V, Stefanou MI, Demetriou M, Siafakas N, Makris M, Tsivgoulis G, et al. Long COVID and neuropsychiatric manifestations (Review). Experimental and Therapeutic Medicine. 2022;23(5):363.

*Reason for exclusion:* Construct not according to a mental health diagnosis

23. Esposito S, Deolmi M, Ramundo G, Puntoni M, Caminiti C, Principi N. True prevalence of long COVID in children: a narrative review. Frontiers in Microbiology. 2023;14:1225952.

*Reason for exclusion:* No mental health diagnosis before Long COVID

24. Fenton C, Lee A. Antidepressants with anti-inflammatory properties may be useful in long COVID depression. Drugs and Therapy Perspectives. 2023;39(2):65-70.

*Reason for exclusion:* Construct not according to a mental health diagnosis

25. Fierini F, Moretti D, Ballerini A. Psychosis spectrum disorders during and after the COVID-19 pandemic: Warning signs of "stress incubation". Psychiatry Research. 2020;291:113291.

*Reason for exclusion:* No Long COVID

26. Frolov L, Jadhav S. 38.1 Clinical Updates on Postacute COVID-19 Sequelae and Considerations for Vulnerable Populations. Journal of the American Academy of Child and Adolescent Psychiatry. 2022;61(10):S52.

*Reason for exclusion:* Wrong publication type

27. Gasnier M, Choucha W, Radiguer F, Faulet T, Chappell K, Bougarel A, et al. Comorbidity of long COVID and psychiatric disorders after a hospitalisation for COVID-19: a cross-sectional study. J Neurol Neurosurg Psychiatry. 2022.

*Reason for exclusion:* No mental health diagnosis

28. Gasnier M, David D, Becquemont L, Corruble E, Colle R. 128. Long Covid and Psychiatric Disorders 4 Months After a Hospitalization for COVID-19: A Cross Sectional Study. Biological Psychiatry. 2023;93(9):S146.

*Reason for exclusion:* Wrong publication type

29. Gasnier M, Montani D, Corruble E, Colle R. Psychiatric disorders and long COVID. Respiratory Medicine and Research. 2022;82:100958.

*Reason for exclusion:* No mental health diagnosis before Long COVID

30. Gonzalez-Fern, ez E, Huang J. Cognitive Aspects of COVID-19. Curr Neurol Neurosci Rep. 2023;23(9):531-8.

*Reason for exclusion:* No mental health diagnosis before Long COVID

31. Goodman ML, Molldrem S, Elliott A, Robertson D, Keiser P. Long COVID and mental health correlates: a new chronic condition fits existing patterns. Health Psychology and Behavioral Medicine. 2023;11(1):2164498.

*Reason for exclusion:* No mental health diagnosis

32. Hazumi M, Usuda K, Okazaki E, Kataoka M, Nishi D. Differences in the Course of Depression and Anxiety after COVID-19 Infection between Recovered Patients with and without a Psychiatric History: A Cross-Sectional Study. Int J Environ Res Public Health. 2022;19(18).

*Reason for exclusion:* No Long COVID

33. Ismael F, Bizario JCS, Battagin T, Zaramella B, Leal FE, Torales J, et al. Post-infection depressive, anxiety and post-traumatic stress symptoms: A prospective cohort study in patients with mild COVID-19. Progress in neuro-psychopharmacology & biological psychiatry. 2021;111:110341-.

*Reason for exclusion:* No Long COVID

34. Janiri D, Carfì A, Kotzalidis GD, Bernabei R, i F, Sani G. Posttraumatic Stress Disorder in Patients After Severe COVID-19 Infection. JAMA psychiatry. 2021;78(5):567-9.

*Reason for exclusion:* No Long COVID

35. Jileaeva I, Bakre S, Huff L, Ratnakaran B. The Same Day on Repeat: A Unique Case of Persistent Deja Vu Phenomenon as a Long COVID Symptom in an Older Adult. European Psychiatry. 2023;66:S793.

*Reason for exclusion:* Wrong publication type

36. Kennelly CE, Nguyen ATP, Sheikhan NY, Strudwick G, Ski CF, Thompson DR, et al. The lived experience of long COVID: A qualitative study of mental health, quality of life, and coping. PLoS One. 2023;18(10):e0292630.

*Reason for exclusion:* No mental health diagnosis

37. Lerer L, Cherney L, Roth E. Characterizing Symptoms and Impact of "Long Covid": A Qualitative Perspective. Archives of Physical Medicine and Rehabilitation. 2022;103(12):e49.

*Reason for exclusion:* Wrong publication type

38. Lerer L, Marcangelo M, Cherney LR, Roth EJ. (30) Characterizing Mood Symptoms in Long Covid: A Qualitative Perspective. Journal of the Academy of Consultation-Liaison Psychiatry. 2022;63:S127.

*Reason for exclusion:* Wrong publication type

39. Marshall MS. The four most urgent questions about long COVID. Nature. 2021;594(7862):168-70.

*Reason for exclusion:* No mental health diagnosis before Long COVID

40. Miller MJ, Feldstein LR, Holbrook J, Plumb ID, Accorsi EK, Zhang QC, et al. Post-COVID conditions and healthcare utilization among adults with and without disabilities-2021 Porter Novelli FallStyles survey. Disability and Health Journal. 2023;16(2):101436.

*Reason for exclusion:* No mental health diagnosis

41. Mir D, a DAP, Gomes SVC, Filgueiras PS, Corsini CA, Almeida NBF, et al. Long COVID-19 syndrome: A 14-months longitudinal study during the two first epidemic peaks in Southeast Brazil. Transactions of the Royal Society of Tropical Medicine and Hygiene. 2022;116(11):1007-14.

*Reason for exclusion:* No mental health diagnosis before Long COVID

42. Molero P, Reina G, Blom JD, Martínez-González M, Reinken A, de Kloet ER, et al. COVID-19 risk, course and outcome in people with mental disorders: a systematic review and meta-analyses. Epidemiol Psychiatr Sci. 2023;32:e61.

*Reason for exclusion:* No Long COVID

43. Omar YO. Development of endurance training program for patient diagnosed with post-COVID syndrome: A case report. Cardiopulmonary Physical Therapy Journal. 2022;33(1):e13-e4.

*Reason for exclusion:* Wrong publication type

44. Padda KS, Shalev D, Thakur K, Edelstein A, Re'em Y. (62) A Case Series: Psychiatric Comorbidity in Long Covid. Journal of the Academy of Consultation-Liaison Psychiatry. 2022;63:S143.

*Reason for exclusion:* No mental health diagnosis

45. Philip S, Shoib S, Gregor Issac T, Javed S. Diagnostic challenges posed by intersections between post-acute covid syndrome and neurocognitive disorders. Asian J Psychiatr. 2022;67:102936.

*Reason for exclusion:* Construct not according to a mental health diagnosis

46. Phu DH, Maneerattanasak S, Shohaimi S, Trang LTT, Nam TT, Kuning M, et al. Prevalence and factors associated with long COVID and mental health status among recovered COVID-19 patients in southern Thailand. PLoS One. 2023;18(7):e0289382.

*Reason for exclusion:* No mental health diagnosis before Long COVID

47. Prato A, Salerno AM, Saia F, Maugeri N, Zanini A, Scerbo M, et al. Symptoms compatible with long COVID in an Italian pediatric cohort of Tourette patients with and without SARS‑CoV‑2 infection: a short-term follow-up assessment. BMC Pediatr. 2023;23(1):222.

*Reason for exclusion:* No Long COVID

48. Rasmus P, Kasprzak JD, Chudzik M, Lipert A. Anxiety and depression among ambulatory and hospitalized patients with cardiovascular long-COVID-19 presentation referred to cardiology consultation after COVID-19 infection. European Respiratory Journal. 2022;60:2426.

*Reason for exclusion:* No mental health diagnosis before Long COVID

49. Rastogi R, Cerda IH, Ibrahim A, Chen JA, Stevens C, Liu CH. Long COVID and psychological distress in young adults: Potential protective effect of a prior mental health diagnosis. Journal of Affective Disorders. 2023;340:639-48.

*Reason for exclusion:* No mental health diagnosis before Long COVID

50. Rawlings GH, Beail N. Long-COVID in people with intellectual disabilities: A call for research of a neglected area. British Journal of Learning Disabilities. 2022;51(1):91-8.

*Reason for exclusion:* Construct not according to a mental health diagnosis

51. Rich SE. PROMIS tool implementation to improve depression screening: A quality improvement project in a covid recovery clinic: ProQuest Information & Learning; 2023.

*Reason for exclusion:* Construct not according to a mental health diagnosis

52. Richter K, Kellner S. "Coronasomnia"-promoting resilience through insomnia treatment. Somnologie. 2021;25(3):170-5.

*Reason for exclusion:* No mental health diagnosis before Long COVID

53. Rouen A, Taïeb J, Caetano G, Pitron V, Elbaz M, Salmon D, et al. Polysomnographic parameters in long-COVID chronic insomnia patients. Dialogues Clin Neurosci. 2023;25(1):43-9.

*Reason for exclusion:* No mental health diagnosis before Long COVID

54. Rudenstine S, Schulder T, Bhatt KJ, McNeal K, Ettman CK, Galea S. Long-COVID and comorbid depression and anxiety two years into the COVID-19 pandemic. Psychiatry Res. 2022;317:114924.

*Reason for exclusion:* No mental health diagnosis before Long COVID

55. Sansone D, Tassinari A, Valentinotti R, Kontogiannis D, Ronchese F, Centonze S, et al. Persistence of Symptoms 15 Months since COVID-19 Diagnosis: Prevalence, Risk Factors and Residual Work Ability. Life (Basel). 2022;13(1).

*Reason for exclusion:* No mental health diagnosis before Long COVID

56. Schulder T, Rudenstine S, Ettman CK, Galea S, ro. Correlates of long-COVID-19: the role of demographics, chronic illness, and psychiatric diagnosis in an urban sample. Psychology, Health & Medicine. 2023;28(7):1831-43.

*Reason for exclusion:* No mental health diagnosis before Long COVID

57. Shakya P, Narnoli S, Surendranath A, Verma R, Mohan A, Sharan P, et al. Rapid screening for psychiatric co-morbidity in patients recovering from Covid-19 infection in a clinic in a low-and middle-income country. General Hospital Psychiatry. 2022;79:185-6.

*Reason for exclusion:* No mental health diagnosis before Long COVID

58. Simonetti A, Bernardi E, Margoni S, Catinari A, Restaino A, Ieritano V, et al. Mixed Depression in the Post-COVID-19 Syndrome: Correlation between Excitatory Symptoms in Depression and Physical Burden after COVID-19. Brain Sciences. 2023;13(4):688.

*Reason for exclusion:* No mental health diagnosis before Long COVID

59. Skilbeck L. Patient-led integrated cognitive behavioural therapy for management of long COVID with comorbid depression and anxiety in primary care - A case study. Chronic Illness. 2022;18(3):691-701.

*Reason for exclusion:* No mental health diagnosis before Long COVID

60. Spurio MG. Long-COVID and Stress Overload: A Direct Link with Psychophysical Disease. A Necessary Interconnection to Focalize Emerging Needs and Necessities. Psychiatr Danub. 2022;34:90-5.

*Reason for exclusion:* No mental health diagnosis before Long COVID

61. Steenblock C, Schwarz PEH, Perakakis N, Brajshori N, Beqiri P, Bornstein SR. The interface of COVID-19, diabetes, and depression. Discover Mental Health. 2022;2(1):5.

*Reason for exclusion:* No mental health diagnosis before Long COVID

62. Simonetti A, Bernardi E, Janiri D, Mazza M, Montanari S, Catinari A, et al. Suicide Risk in Post-COVID-19 Syndrome. Journal of Personalized Medicine. 2022;12(12):2019.

*Reason for exclusion:* Construct not according to a mental health diagnosis

63. Strawn J, Mills J, Schroeder H, Neptune Z, Specht A, Keeshin S. Severe Acute Respiratory Syndrome Coronavirus-2 (SARS-CoV-2) Infection and "Long COVID" in Adolescents With Anxiety Disorders: A Prospective Longitudinal Study. Neuropsychopharmacology. 2022;47:106-7.

*Reason for exclusion:* Wrong publication type

64. Tak CR, gren K, Hughes P, Ramage M. Long COVID and Mental Health: An Assessment of Health Status and Service Utilization. Journal of Mental Health Policy and Economics. 2023;26:S31.

*Reason for exclusion:* No mental health diagnosis before Long COVID

65. Talhari C, Criado PR, Castro CCS, Ianhez M, Ramos PM, Miot HA. Prevalence of and risk factors for post-COVID: Results from a survey of 6,958 patients from Brazil. An Acad Bras Cienc. 2023;95(1):e20220143.

*Reason for exclusion:* No mental health diagnosis before Long COVID

66. Tarnanas I, Tsolaki M. Making pre-screening for Alzheimer's disease (AD) and Postoperative delirium among post-acute COVID-19 syndrome - (PACS) a national priority: The Deep Neuro Study. Open Res Eur. 2022;2:98.

*Reason for exclusion:* Wrong publication type

67. Tarnanas I, Tsolaki M. Making Pre-screening for Alzheimer's Disease (AD) and Postoperative Delirium Among Post-Acute COVID-19 Syndrome (PACS) a National Priority: The Deep Neuro Study. Adv Exp Med Biol. 2023;1424:41-7.

*Reason for exclusion: Duplicate*

68. Thawani S, Kenney R, Hasanaj L, Frontera J, Seixas A, Galetta S, et al. The post-acute sequelae of COVID-19 (PASC) experience in an outpatient neurology setting. Neurology. 2022;98(18).

*Reason for exclusion:* Wrong publication type

69. Thurner C, Stengel A. Long-COVID syndrome: physical-mental interplay in the spotlight. Inflammopharmacology. 2023;31(2):559-64.

*Reason for exclusion:* No mental health diagnosis

70. Tizenberg BN, Brenner LA, Lowry CA, Okusaga OO, Benavides DR, Hoisington AJ, et al. Biological and Psychological Factors Determining Neuropsychiatric Outcomes in COVID-19. Curr Psychiatry Rep. 2021;23(10):68.

*Reason for exclusion:* Construct not according to a mental health diagnosis

71. Valenza M, Steardo L, Verkhratsky A, Scuderi C. Systemic Inflammation and Astrocyte Reactivity in the Neuropsychiatric Sequelae of COVID-19: Focus on Autism Spectrum Disorders. Frontiers in Cellular Neuroscience. 2021;15:748136.

*Reason for exclusion:* No mental health diagnosis before Long COVID

72. Vélez M, Falconí Paez A, Nicolalde B, Esquetini-Vernon C, Lara-Taranchenko Y, Zambrano K, et al. Cognitive impairment or dementia in post-acute COVID-19 syndrome. Two suspects and a perfect detective: Positron emission tomography (PET) scan. European Neuropsychopharmacology. 2022;61:91-3.

*Reason for exclusion:* Wrong publication type

73. Villalp, o JMG, Forcelledo HA, Castillo JLB, Sastré AJ, Rojop IEJ, et al. COVID-19, Long COVID Syndrome, and Mental Health Sequelae in a Mexican Population. Int J Environ Res Public Health. 2022;19(12).

*Reason for exclusion:* No mental health diagnosis before Long Covid

74. Walia N, Lat JO, Tariq R, Tyagi S, Qazi AM, Salari SW, et al. Post-acute sequelae of COVID-19 and the mental health implications. Discoveries (Craiova). 2021;9(4):e140.

*Reason for exclusion:* No mental health diagnosis before Long COVID

75. Woodward JM, Liu TL, Kowalkowski M, Taylor YJ, Gutnik B, Mangieri DA. Assessing post-COVID symptomatology among persons with dementia and other older adults who were hospitalized due to COVID-19: An observational study. Health Sci Rep. 2023;6(7):e1345.

*Reason for exclusion:* Construct not according to a mental health diagnosis

76. Xiao Y, Sharma MM, Thiruvalluru RK, Gimbrone C, Weissman MM, Olfson M, et al. Trends in psychiatric diagnoses by COVID-19 infection and hospitalization among patients with and without recent clinical psychiatric diagnoses in New York city from March 2020 to August 2021. Transl Psychiatry. 2022;12(1):492.

*Reason for exclusion:* No Long COVID

77. Zhang D, Chung VC, Chan DC, Xu Z, Zhou W, Tam KW, et al. Determinants of post-COVID-19 symptoms among adults aged 55 or above with chronic conditions in primary care: data from a prospective cohort in Hong Kong. Front Public Health. 2023;11:1138147.

*Reason for exclusion:* Construct not according to a mental health diagnosis
